# Intermittent Fever

**Published:** 1874-05

**Authors:** 


*Intermittent Fever.*—It is allowable for the patient, after admission, to have one chill, in order to determine the character of his disease; but subsequent to that the occurrence of chills is not permitted. The certain arrest of the case is looked for under the influence of what are denominated Clark's powders, which are formulated as follows:—

| | |
|---|---|
| ℞.—Quiniæ sulphat, . . . . . | grs. x. |
| Pulv. capsici, . . . . . | grs. iij. |
| Pulv. opii., . . . . . . | gr. j.—M. |

The patient receives one powder twelve hours previous to the time for the occurrence of the chill, and another about an hour previous.

The powders are continued in this manner for three or four days, and then continued every morning as long as may be desired. An important consideration affecting the continuation of the remedy is the following: It not infrequently happens that the chills return after they have been stopped, and the most common time for them to return is at the end of two weeks. Near the expiration of two weeks, therefore, it is well to return to the full-sized powders, if they have been diminished in size, and continue them in full doses for three or four days, twice a day, as at the outset. In cases which have been of long standing, and proved themselves very intractable, when the chills have been stopped the remedy should be continued over three or four periods of return at least. By this method of treatment cases of chills and fever can be cured which have resisted all former treatment.

After the breaking of the chill the administration of iron is about as useful as the administration of quinine before. If the second chill occurs after admission to the hospital, the question, Why? is asked at once, and search made for the answer.

